# Fisher (*Pekania pennanti*) Populations Exhibit Regional Differences in Cause‐Specific Mortality but Not Survival Rates

**DOI:** 10.1002/ece3.71531

**Published:** 2025-06-05

**Authors:** Justin J. Remmers, Kirk W. Stodola, Maximilian L. Allen

**Affiliations:** ^1^ Illinois Natural History Survey Prairie Research Institute, University of Illinois Champaign Illinois USA

**Keywords:** conservation, demography, mortality, *Pekania pennanti*, survival

## Abstract

Mortality causes and survival rates often vary between the geographically disparate populations of a species. Fishers ( *Pekania pennanti* ) are a mesocarnivore inhabiting forested areas across Canada and the United States of America. Due to their economic and ecological value, fishers have become the focus of many management and conservation efforts. However, a clear understanding of influential demographic parameters and pressures exerted on disparate populations is necessary for such discussions. We conducted a literature review of peer‐reviewed studies investigating fisher cause‐specific mortalities and survival to (a) synthesize the current available knowledge, (b) assess differences in cause‐specific mortalities and the sex‐specific adult survival rates between western fisher populations (i.e., populations from California, Oregon, Washington, or British Columbia) and eastern fisher populations (i.e., elsewhere in their distribution), and (c) identify potential gaps in the literature. We identified 26 studies between 1994–2024 describing cause‐specific mortality (*n* = 4), survival rates (*n* = 15 studies), or both (*n* = 7), with 20 studies assessing western fisher populations. There were significant differences between the cause‐specific mortalities for fishers in the eastern and western populations. Western fishers had higher mortality from predation and lethal toxicant exposure, while eastern fishers had higher mortality from legal harvest. Survival rates of males and females were not significantly different between the eastern and western populations; however, we found that male survival rates in the western populations varied considerably between studies. The geographic concentration of recent research presents a lack of information regarding the species outside of western populations, which may hinder management efforts throughout their range.

## Graphical abstract

Western fisher populations have been the focus of many recent studies and exhibit differences in cause‐specific mortalities but not in survival rates when compared to eastern fisher populations. Western fisher mortalities were dominated by predator attacks while eastern fisher mortalities were dominated by harvest.

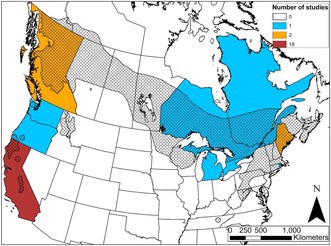

## 1 Introduction

Survival rates frequently vary depending on the unique pressures experienced by the disparate populations of a species (Krebs et al. 2004; Rodewald and Gehrt 2014), which are often characterized as differing mortality sources (Collins and Kays 2011; Hill et al. 2019). Anthropogenic pressures (e.g., harvest, toxicant exposures, vehicular strikes; Krohn et al. 1994, Koen et al. 2007, Gabriel et al. 2015), natural causes (e.g., disease, starvation; Bender et al. 2004, Hill et al. 2019), and interspecific interactions (e.g., predation, competition; Collins and Kays 2011, Wengert et al. 2014) are common causes of mortality in wildlife populations. However, exposure to any individual mortality source depends on the behavior and geographic location of a specific population (Collins and Kays 2011; Rodewald and Gehrt 2014; Hill et al. 2019). This creates challenges for managers and conservation efforts focused on species whose mortality sources may drastically differ across their distribution. Previously published research may not accurately reflect the current pressures influencing a given population (Clark and Fritzell 1992; Munns 2006), complicating comparison among studies or necessitating assumptions be made about understudied populations.

Fishers ( *Pekania pennanti* ) are a medium‐sized carnivore that inhabit forests across multiple political boundaries, ecoregions, and physiographic regions in Canada and the United States of America (Powell 1993). During the late 1800s and early 1900s, habitat loss, predator control measures, and overharvest by fur trappers, driven by the high demand for fisher fur, resulted in fisher population declines and range contractions. Throughout this period, fishers were extirpated from much of their historic range (Powell et al. 2003). In response to declining fisher populations, many states ended fisher harvest, instituted legal protections, or began management efforts (Powell 1993; Powell and Zielinski 1994; Lewis et al. 2012). Such protections and efforts allowed fishers to recolonize parts of their historic range (Coulter 1960; Aubry and Lewis 2003; Allen et al. 2024) and dramatically increase their distribution (Lewis et al. 2012). To further bolster their recovery, there have been at least 35 fisher translocation efforts undertaken by 14 states and 6 Canadian provinces between 1947 and 2004, with most considered successful (Lewis 2006; Lewis et al. 2012). The recovery of fishers prompted discussion regarding the value of the species as a natural resource and led to many midwestern and northeastern states reinstating legal harvest (Powell 1993). More recently, there have been a slew of reintroduction projects focused on fisher populations in California and Washington (e.g., Green, Facka, et al. 2022; Lewis et al. 2022). As the populations in these areas are still recovering, legal harvest is prohibited throughout much of that region.

Yet much debate remains surrounding the current status of fishers. Researchers observed the potential for increased fisher mortality or harvest leading to local extinction events (Spencer et al. 2011; Buskirk et al. 2012; Fogarty et al. 2022) and raised concerns over the impacts of forest management and agricultural pesticides on fishers (Matthews et al. 2013; Sweitzer et al. 2015). Furthermore, western fisher populations (i.e., California, Oregon, Washington, and British Columbia) were recently under consideration for legal protection through the Endangered Species Act (United States Fish and Wildlife Service [USFWS] 2020). Conversely, eastern fisher populations (i.e., fisher populations elsewhere throughout their distribution) are typically subject to legal harvest of fishers; however, harvest levels and regulations vary considerably between jurisdictions (Powell et al. 2003).

Given that fishers occur across a wide and varied geographic area, the pressures faced by individual populations likely also differ. Indeed, fisher vital rates are distinctly different between populations and geographic regions, causing profound changes to population growth (Buskirk et al. 2012; Lofroth et al. 2023). Furthermore, Green, Purcell, et al. (2018) found that reproductive parameters differed between studies, with the proportion of female fishers reproducing ranging from 0.40 to 1.00 and mean litter sizes ranging from 1.57 to 3.4, and attributed differences to study methods, study population (e.g., captive‐bred, wild‐caught), geographic location, and climatic conditions. Other researchers posited prey availability (Bulmer 1974), habitat quality (Powell and Zielinski 1994), or harvest pressure (Kelly 1977) as alternative explanations for changes in fisher vital rates. Such differences in study methods, target populations, geographic regions, or environmental pressures may similarly influence reports of cause‐specific mortalities or survival rates. Given that survival is more influential on fisher population growth than reproduction (Buskirk et al. 2012), further examination of current literature on the topic is warranted. Thus, the goals of this paper were to (1) provide a range‐wide review and synthesis of available peer‐reviewed scientific literature on fisher cause‐specific mortality and survival rates, (2) assess differences in causes of mortalities and survival rates between the western and eastern fisher populations, and (3) identify geographic or topical gaps in the literature for future research.

## 2 Methods

### 2.1 Literature Review

On 15 November 2024, we performed a systematic literature search to determine the frequency of documentation of cause‐specific mortality reports and survival rates among fisher populations. We searched Web of Science for the terms “ *Martes pennanti* ,” “ *Pekania pennanti* ,” and “fisher” “pennanti” matched with the search terms “survival,” “mortality,” or “vital rates” in English. We then assessed the full text of each entry and removed duplicate reports and mismatched publications (e.g., captive studies, studies on density or home range). We augmented our systematic literature review using snowball sampling (i.e., searching the references of papers we reviewed for additional documentation). We only considered literature from published, peer‐reviewed journal articles or book chapters. We examined each study for information regarding reports of cause‐specific mortality or survival rates. For cause‐specific mortality, we considered any studies regardless of original purpose or study design that contained reports of at least 1 fisher mortality with a confirmed cause of death. For survival rates, we only considered studies that reported survival rates from observational studies (e.g., radiotelemetry) or as derived parameters in modeling efforts (e.g., apparent survival). We also documented the geographic location of the study area and sample size whenever possible.

### 2.2 Statistical Analysis

We compared the number of reports of cause‐specific mortalities between studies examining the western and eastern populations using a chi‐squared test. We organized cause‐specific mortality into five categories: harvest (i.e., legal harvest, illegal harvest, incidental harvest), toxicant (i.e., lethal poisoning), other human (e.g., vehicular strike, entrapment in human structures), natural (e.g., predation, starvation, drowning, disease), or unknown. We used a chi‐squared test to compare each category individually and among all categories, excluding unknown cause. We ensured that all expected frequency values were > 1.

We obtained survival estimates for adult fishers from literature gathered during the review process. Whenever possible, we obtained annual survival rates for both adult males and females. In cases when only a single annual survival rate was reported by a study, we assigned the reported value to both males and females as a “combined” annual survival rate. If a study reported seasonal rates (e.g., summer, harvest), we estimated annual survival by assuming the interaction of the survival rates for each reported season. In some studies, multiple annual survival rates were given dependent on an experimental factor (e.g., Kordosky et al. 2021). In these cases, we used the mean adult sex‐specific survival rate from the multiple rates reported. When annual survival rates were given for multiple years, we included all annual values. We conducted Mann Whitney tests to assess differences between survival rates between (1) sex‐specific reports for all male and female fishers, (2) female fishers from the western and eastern populations, and (3) male fishers from the western and eastern populations. When comparing western and eastern populations, we conducted two versions of the Mann Whitney tests: (1) using only sex‐specific survival estimates and (2) using sex‐specific and combined rates assigned to both sexes.

Additionally, we obtained survival rates for young fishers whenever possible. We considered young fishers to be any category not classified as an adult by the original study and included fishers aged as juveniles (e.g., > 1 year) or subadults (e.g., 1–2 years) in this group. In all statistical analyses, we used *α* = 0.05 to assess significance. We conducted all statistical analyses in R version 4.3.3 (R Core Team 2024).

## 3 Results

**FIGURE 1:**
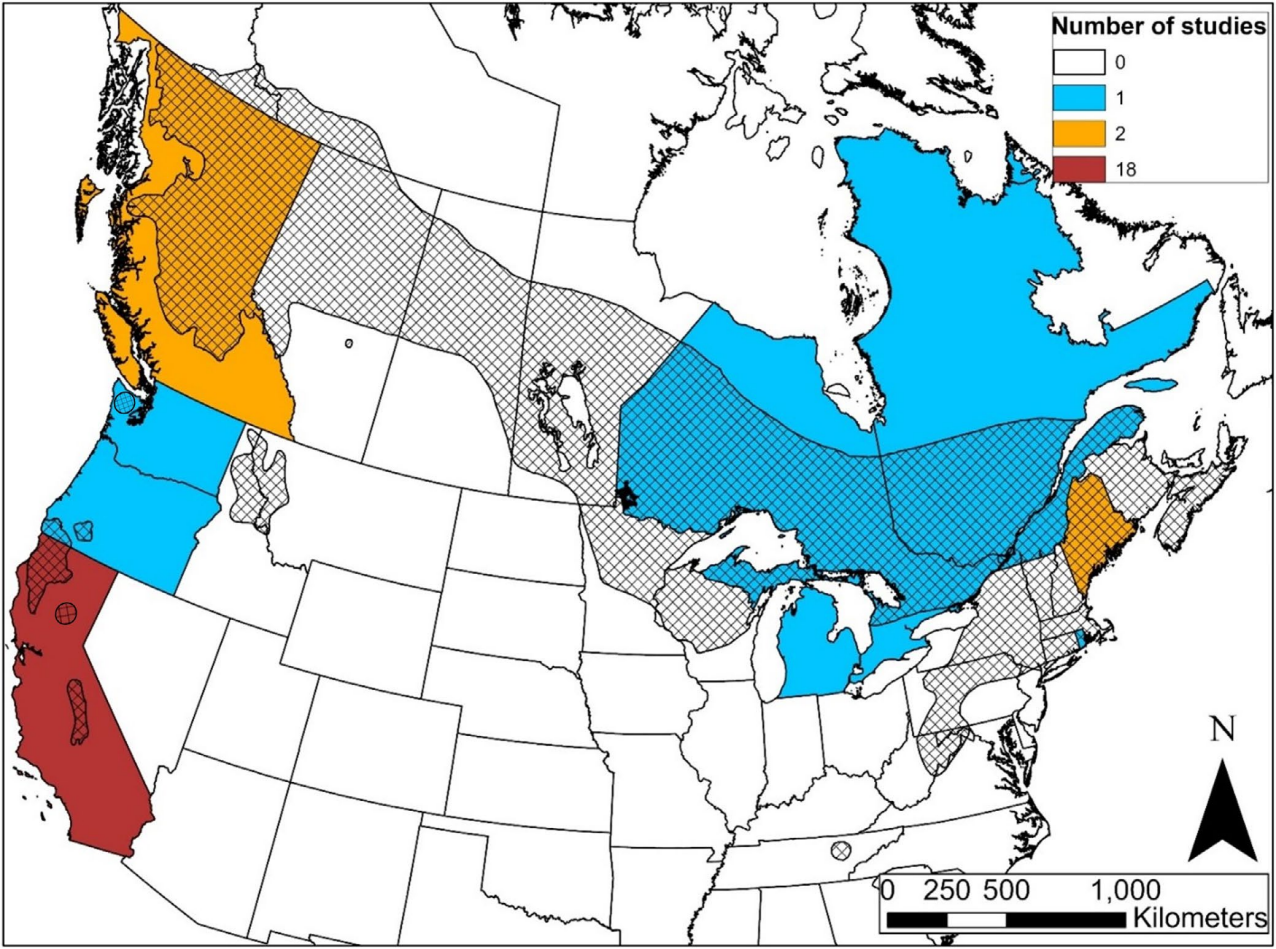
Geographic distribution of published peer‐reviewed studies on fisher survival rates and cause‐specific mortality. Hatched areas represent the current distribution of fishers based on Helgen and Reid (2018) with additions to represent the introduced populations described by Lewis et al. (2022) and Green, Facka, et al. (2022).

**FIGURE 2:**
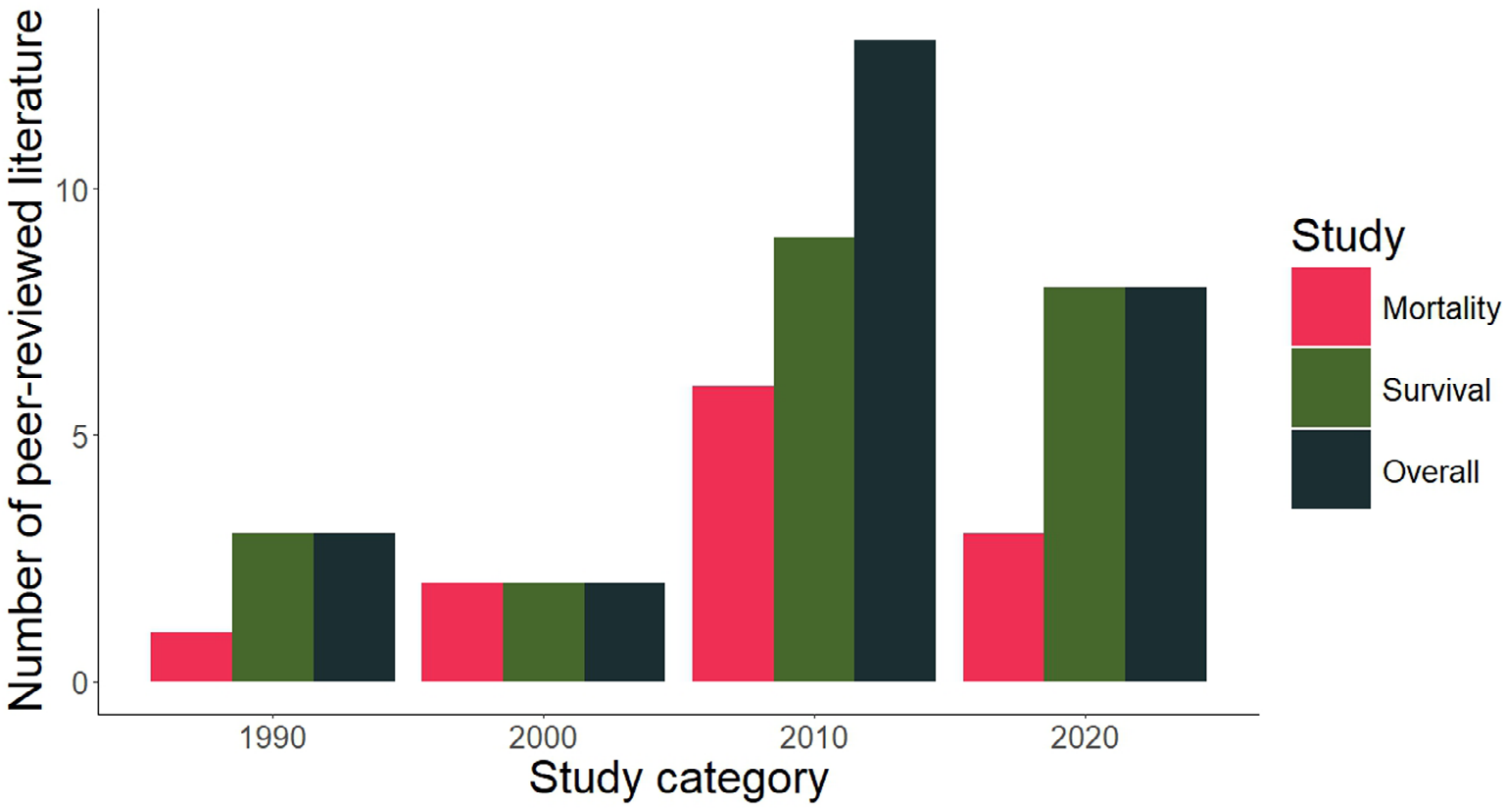
Number of peer‐reviewed studies identified by the literature review about fisher survival rates and cause‐specific mortality by decade. We gathered studies through a literature review conducted on the Web of Science, November 15, 2024.

Our search in Web of Science returned an initial list of 75 peer‐reviewed journal articles. After expanding the potential literature pool through snowball sampling and removal of mismatched sources, we identified 26 peer‐reviewed articles or book chapters that fit our search criteria. Of these, 4 contained cause‐specific reports of morality, 15 contained fisher survival rates, and 7 contained both survival rates and cause‐specific morality (Table S1). Although studies were conducted across the geographic range of fishers, most studies occurred in coastal states or provinces (Figure 1). Studies occurred between 1994–2024, with most studies happening during the 2010s (50%) and 2020s (30%; Figure 2), potentially due to scientific advances enabling cost‐effective tracking of individuals as well as increased interest in the survival of translocated populations throughout western populations.

### 3.1 Causes of Mortality

**FIGURE 3:**
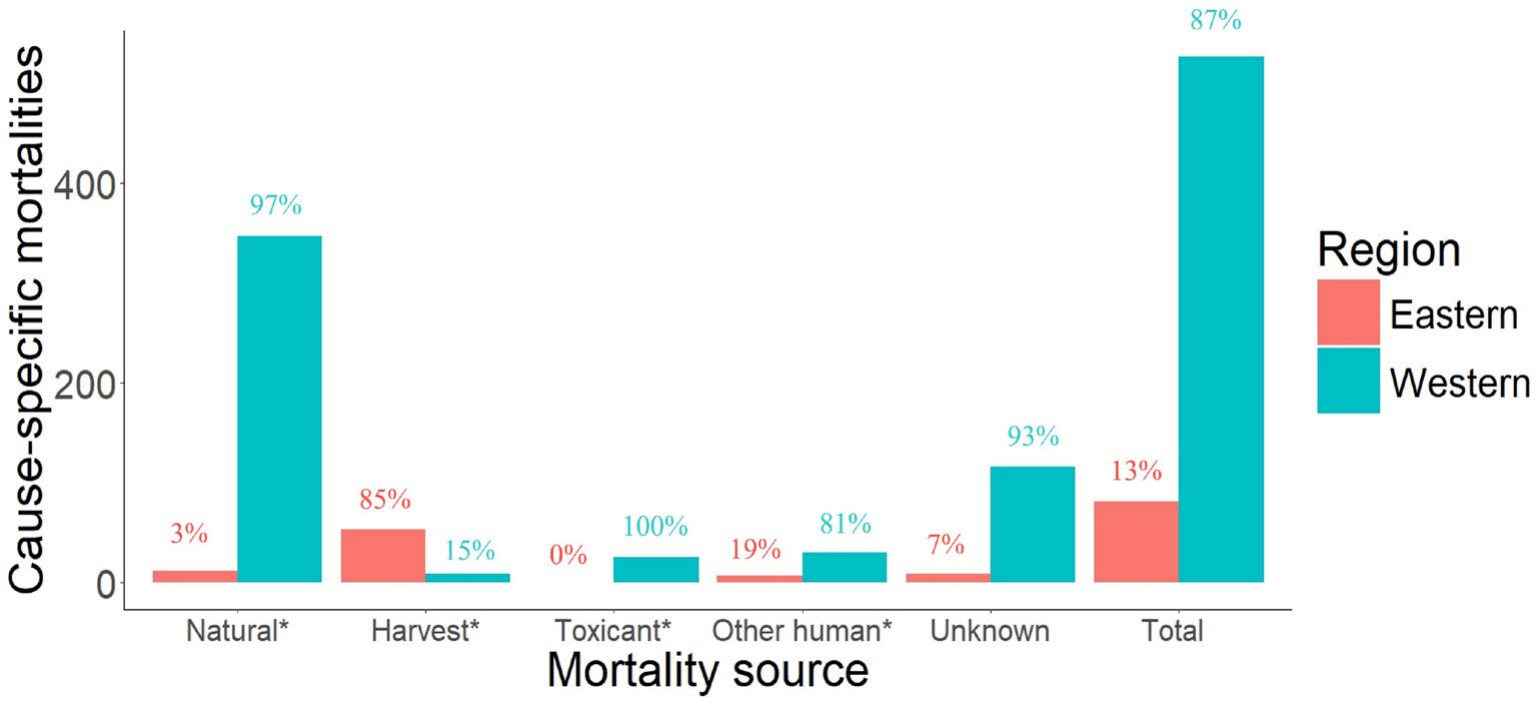
Number of cause‐specific mortalities reported in peer‐reviewed literature for the western fisher populations (i.e., populations from California, Oregon, Washington, or British Columbia) and eastern fisher populations (i.e., populations from elsewhere in their distribution). Cause‐specific mortalities were classified as natural (e.g., predation, starvation, disease), harvest (i.e., legal harvest, illegal harvest, incidental harvest), toxicant (i.e., lethal poisoning), other human (e.g., vehicular strike, entrapment in human structures), or unknown. Proportion of total cause‐specific mortalities reports per classification occurring in each region are reported above each column and the classifications with significance differences between the number of reports from each region is represented by an asterisk (*) next to the classification title. We gathered reports through a literature review conducted on the Web of Science, November 15, 2024.

We identified 12 studies with cause‐specific mortality reports (Table S1). We observed 483 reports of cause‐specific mortalities, discounting the 125 mortalities with unknown causes, across the distributional range of fishers (Table 1, Figure 3). Mortalities were most often from natural causes (59.5%), followed by unknown causes (20.0%) and then harvest (10.2%), while other human causes (6.1%) and toxicant (4.1%) were the least common (Figure 3). Of the 10 studies reporting natural causes of mortalities, eight provided additional details regarding the specific mortality source. Predation was the most common natural mortality source with 290 reported cases (80% of total natural cause mortality reports). Starvation and disease were the second most common natural mortality sources with 49 reported cases (14% of total natural cause mortality reports).

**TABLE 1:** Cause‐specific mortality as reported by studies identified in the literature review.

Source	Location	Mortality source				
		Harvest	Toxicant	Other human	Natural	Unknown
Gabriel et al. (2012)	California	—	4	—	—	—
Matthews et al. (2013)	California	—	—	—	1	5
Thompson et al. (2014)	California	—	1	—	45	—
Wengert et al. (2014)	California	—	—	—	62	39
Gabriel et al. (2015)	California	—	13	12	115	27
Sweitzer, Thompson, et al. (2016)	California	—	—	1	7	—
Sweitzer, Popescu, et al. (2016)	California	—	6	10	89	30
Lewis et al. (2022)	Washington	1	—	7	16	11
Lofroth et al. (2023)	British Columbia	8	1	—	12	4
Krohn et al. (1994)	Maine	43	—	4	4	1
Belant (2007)	Michigan	1	—	—	—	—
Koen et al. (2007)	Ontario	9	—	3	8	8
Total	—	62	25	37	359	125

*Note:* Mortality sources were classified as harvest (i.e., incidental harvest, fur trapping harvest), toxicant, other human (e.g., vehicular strike, entrapment in human structures), natural (e.g., predation, starvation, disease), or unknown. Studies investigating eastern fisher populations are shaded in gray at the bottom of the table.

We found that there were significant differences between western and eastern fisher populations in all measured categories of mortality (*x* ^2^ = 286.8, df = 3, *p* < 0.0001). Between eastern and western populations, there were significant differences in reports of cause‐specific mortalities, with natural cause mortalities (*n* = 359, *x* ^2^ = 313.6, df = 1, *p* < 0.0001) the most reported, followed by harvest mortalities (*n* = 62, *x* ^2^ = 31.2, df = 1, *p* < 0.0001), other human mortalities (*n* = 37, *x* ^2^ = 14.3, df = 1, *p* < 0.0001), and toxicant mortalities (*n* = 25, *x* ^2^ = 25.0, df = 1, *p* = 0.002). Western populations reported more mortalities due to natural causes, toxicants, and other human sources, while the eastern population had more mortality reports from harvest.

### 3.2 Survival Rates

**FIGURE 4:**
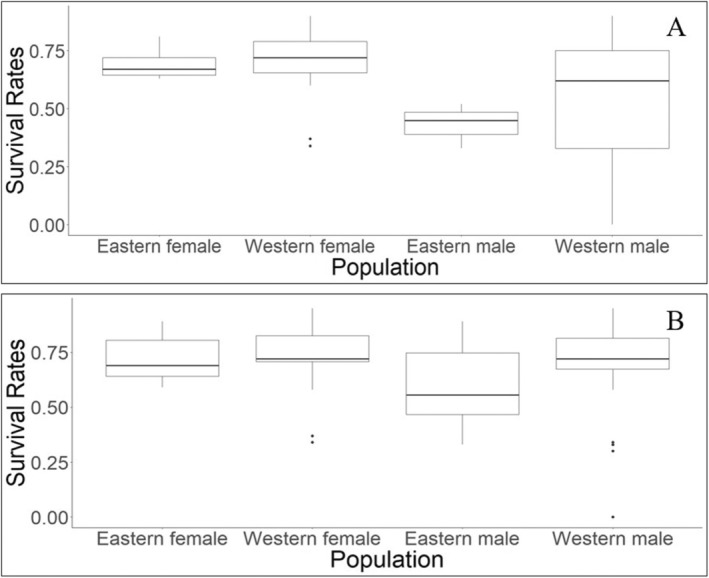
Distribution of reported survival rates for adult male and female fishers in the western populations (i.e., California, Oregon, Washington, British Columbia) and eastern populations (i.e., elsewhere throughout the rest of the fisher distribution in North America) when only sex‐specific survival rates were considered (A) and when sex‐specific and combined survival rates were considered (B). We gathered studies through a literature review conducted on the Web of Science, November 15, 2024.

We identified 13 studies that reported sex‐specific adult survival rates and 9 studies that reported a combined adult survival rate (Table 2). Survival rates varied between populations, ranging from 0.00–0.95 and 0.34–0.84 for adult males and adult females, respectively (Table 2). Studies with sex‐specific adult survival rates generally found males had lower survival estimates compared to female fishers. Adult male fishers in the western populations exhibited the highest degree of variation among survival estimates reported in both the sex‐specific and combined rate assessments (Figure 4). Despite differences in the reported rates for adult males and females, the Mann Whitney test using all sex‐specific rates did not find a significant difference between male and female adult fishers (*w* = 57.5; *p* = 0.12). The Mann Whitney tests did not find a significant difference between the western and eastern populations survival rates of adult females (*w*_sex‐specific only_ = 29, *p*_sex‐specific only_ = 0.86; *w*_with combined_ = 19, *p*_with combined_ = 0.74). While neither Mann Whitney test examining male adult fisher survival rates was significant, we did note a lower *p* value for the sex‐specific Mann Whitney test (*w*_sex‐specific only_ = 29, *p*_sex‐specific only_ = 0.09; *w*_with combined_ = 10.5, *p*_with combined_ = 0.64).

**TABLE 2:** Survival rates from published peer‐reviewed journal articles and book chapters with the corresponding demographic category (i.e., age and sex), time period (i.e., the length of time this survival estimate encompasses), and location of the study. Time periods were classified as annual (encompassing the full year), seasonal (encompassing part of the year), or combined seasonal (the interaction between multiple reported seasonal estimates). When multiple rates for a single demographic category were reported, the range of the reported survival rates was listed. Studies investigating eastern fisher populations are shaded in gray at the bottom of the table.

Study	Location	Time period	Adult			Young		
			Female	Male	Combined	Female	Male	Combined
Jordan et al. (2011)	California, USA	Annual	—	—	0.93–0.95	—	—	—
Spencer et al. (2011)	California, USA	Annual	0.9	—	—	0.5–0.7	—	—
Thompson et al. (2014)	California, USA	Annual	0.72	—	—	—	—	—
Sweitzer et al. (2015)	California, USA	Combined seasonal/annual	—	—	0.72	—	—	0.57–0.72
Sweitzer, Thompson, et al. (2016)	California, USA	Seasonal/annual	0.72	0.62	—	—	—	—
Sweitzer, Popescu, et al. (2016)	California, USA	Seasonal/annual	0.72	—	0.69	0.62	0.57	—
Green, Matthews, et al. (2018)	California & Oregon, USA	Annual	—	—	0.72–0.85	—	—	—
Porteus et al. (2018)	California, USA	Annual	0.34–0.37	0.30–0.34	—	—	—	—
Matthews et al. (2019)	California, USA	Seasonal	—	—	—	0.9–1.0	—	—
Kordosky et al. (2021)	California, USA	Annual	0.84	0.75	—	—	—	—
Green, Facka, et al. (2022)	California, USA	Annual	—	—	0.71	—	—	—
Green, Martin, et al. (2022)	California, USA	Annual	—	—	0.58–0.71	—	—	—
Lewis et al. (2022)	Washington, USA	Annual	0.60–0.71	0.0–0.33	—	0.61–0.67	0.83–0.90	—
Fogarty et al. (2022)	British Columbia, CA	Annual	0.71	0.90	—	0.60	0.86	—
Lofroth et al. (2023)	British Columbia, CA	Annual	0.72	0.72	—	—	—	0.64
Kuntze et al. (2024)	California, USA	Annual	0.76	0.80	—	0.83	0.64	—
Krohn et al. (1994)	Maine, USA	Combined seasonal	0.69	0.52	—	0.28	0.26	—
Paragi et al. (1994)	Maine, USA	Annual	0.65	—	—	—	—	0.27
Garant and Crête (1997)	Quebec, CA	Annual	—	—	0.80	—	—	—
Belant (2007)	Michigan, USA	Combined seasonal	—	—	0.89	—	—	—
Koen et al. (2007)	Ontario, CA	Annual	0.63–0.81	0.33–0.45	—	—	—	—
Bellier et al. (2024)	Rhode Island, USA	Annual	—	—	0.59	—	—	—

We identified 11 studies that reported survival rates for young fishers with estimates ranging from 0.26 to 1.0 (Table 2). Young fisher survival rates were typically lower than adult survival rates. We identified eight studies reporting lower survival rates for young fishers, one estimating higher survival rates for young fishers, and one providing both higher and lower survival estimates for young fishers compared to adult fisher survival. Of the studies reporting lower survival rates for young fishers, differences between the juvenile and adult survival rates ranged from 0.00 (Sweitzer et al. 2015) to 0.41 (Krohn et al. 1994). Only Lewis et al. (2022) and Kuntze et al. (2024) reported any estimates of survival rates higher for young fishers than adult fishers.

We identified eight studies that investigated seasonal survival in some capacity. Krohn et al. (1994) and Belant (2007) indicated that harvest seasons contained lower survival rates than nonharvest seasons for both young and adult fishers. Studies examining biologically significant seasons (e.g., kits while weaning, summer after weaning) found young fishers had lower survival rates than adults (Sweitzer et al. 2015; Sweitzer, Popescu, et al. 2016). Sweitzer, Thompson, et al. (2016) found that fisher survival was lower in mid‐summer compared to fall or winter.

## 4 Discussion

Fishers are an important ecological component and a valuable natural resource (Powell 1993; Powell et al. 2003). While fishers have been the focus of management and conservation efforts (Powell 1993; Powell and Zielinski 1994; Lewis et al. 2012), clear insights into the mechanisms affecting their mortality and survival are necessary to understand population dynamics and craft effective policy. However, our literature review revealed that studies investigating fisher mortality and survival are relatively recent and geographically concentrated (California constituted > 60% of studies). A recent debate over legal protections for western fisher populations under the Endangered Species Act (USFWS 2020) likely attracted widespread interest in these populations, whereas eastern populations have received comparatively less focus (but see Bellier et al. 2024). Consequently, our understanding of current fisher population dynamics is largely based on western populations, leaving managers with limited information to assess the impacts of different harvest regimes and environmental pressures on fisher survival, population trends, and overall ecology in other regions.

### 4.1 Cause‐Specific Mortality

Cause‐specific mortality tended to be from harvest in areas with legal harvest, and natural causes in areas without legal harvest. Studies of populations with some form of legal harvest (i.e., eastern populations) generally observed harvest as the main mortality source (Krohn et al. 1994; Belant 2007; Koen et al. 2007). Given that harvest is not legal in California, Oregon, Washington, and parts of British Columbia, it is unsurprising that little harvest mortality was reported in western populations. Instead, natural causes (e.g., starvation, disease, or predation) were the most commonly reported source of mortality for western fishers. The variation in cause‐specific mortality reports between populations was likely influenced by the differing management approaches adopted by states and provinces across the fisher distribution, with some emphasizing efforts aimed at recolonization while others focus on utilizing fishers as a natural resource. Furthermore, the recent debates over providing legal protections under the Endangered Species Act (USFWS 2020) and reintroduction efforts (Aubry and Lewis 2003; Facka and Powell 2021) may have attracted disproportionate focus on western populations and thus provided more opportunities for researchers to report on fisher mortalities.

Additional geographic disparities may be reflecting differences in predation risks faced by fisher populations across their distribution. Bobcats ( *Lynx rufus* ) and mountain lions ( *Puma concolor* ) were responsible for the majority of reported predation events, along with less frequent predation from coyotes ( *Canis latrans* ) (Wengert et al. 2014; Gabriel et al. 2015). However, mountain lions only overlap fisher distributions in the western regions of North America (Pierce and Bleich 2003; Hornocker and Negri 2009), and the lack of fisher mortalities from predators in eastern populations may be reflective of lower predation risks due to the absence of mountain lions in these areas. Fishers with smaller body sizes (e.g., in more southern areas, like California; Lofroth et al. 2010) are also more prone to predation. This may also explain why predation was less often reported in the eastern populations where fishers have larger mean body sizes for both sexes (Wright and Coulter 1967; Lofroth et al. 2010). Furthering this point was the correlation between predation events and the sexual dimorphism within fishers. The larger male fishers were primarily killed by mountain lions with fewer mortalities from bobcats, whereas the smaller fishers were more often killed by bobcats (Wengert et al. 2014). Thus, differences in fisher mortalities from predation may be due to an overall decreased predation risk by eastern populations due to the absence of larger predators and larger mean body sizes. Alternatively, LaPoint et al. (2014) posited that geographic differences in fisher body sizes arose as fishers evolved to more effectively pursue larger prey in the wake of the mesopredator release as opposed to a defense against predators. Such competing hypotheses emphasize the limits of our current knowledge for this species.

There are additional anthropogenic‐related mortality sources beyond harvest across the fisher range, especially vehicle strikes (Krohn et al. 1994; Koen et al. 2007; Gabriel et al. 2015; Lewis et al. 2022). Previous mammal research found that dispersing or young individuals are more at risk of vehicle strikes (Grilo et al. 2009; Johnson et al. 2010), and young or dispersing fishers also likely experience a greater mortality risk from vehicles. Additional sources of anthropogenic mortality (e.g., handling by researchers, strangulation via radio collar, trapped in cisterns, interspecific interactions with domestic pets; Gabriel et al. 2015, Lewis et al. 2022) were observed as well. While not as prevalent as other mortality sources, these reports highlight how various anthropogenic influences can cause fisher mortalities as well, including rare events from scientific studies.

One additional anthropogenic mortality source of note was lethal exposure to anticoagulant rodenticides or insecticides (Gabriel et al. 2012, 2015; Thompson et al. 2014; Sweitzer, Thompson, et al. 2016; Lofroth et al. 2023). While research investigating exposure to toxic substances (Thomas et al. 2017; Silveira, Frair, Murphy, et al. 2024) or linking toxicant exposure to population trends (Silveira, Frair, Cohen, et al. 2024) has been conducted across multiple geographic regions, we did not identify any confirmed cases of lethal toxicosis outside of western populations, despite researchers finding a high prevalence of toxicant exposure in eastern populations (Buckley et al. 2023). This may be because the main source of toxicant exposure in western populations was associated with marijuana cultivation, which is most prevalent in California (Gabriel et al. 2012; Thompson et al. 2014); while other regions like the Northeast United States were associated with residential use of anticoagulant rodenticides (Silveira, Frair, Murphy, et al. 2024). Marijuana cultivation often occurs in the early spring, when fishers have increased activity, and typically involves the use of large quantities of toxicants in fisher habitat (Gabriel et al. 2015). Indeed, female fisher survival was influenced by the number of marijuana cultivation sites within their home range (Thompson et al. 2014). However, the research regarding the exact risk toxicant exposure poses to fishers, even in California, is conflicting. Sweitzer, Popescu, et al. (2016) found that fishers had a 0.98 survival chance against toxicants, compared to a 0.79 chance against predation. Conversely, Gabriel et al. (2015) reported that male fishers were 13 times more likely than female fishers to die from toxicant exposure than from predation. Male fishers in particular appear to be disproportionately at risk of toxicant exposure, potentially due to larger ranges increasing the likelihood of encountering toxic substances from pesticides or insecticides (Gabriel et al. 2015). As such, the mortality risk from toxicants may depend on multiple factors, including the level of exposure, the type of toxicant, or fisher demography.

### 4.2 Survival

While researchers observed sex‐specific differences in fisher survival rates (Sweitzer et al. 2015; Krohn et al. 1994; Koen et al. 2007; Kordosky et al. 2021), we did not find a significant difference between the overall survival estimates for adult male and female fishers. This was likely influenced by the varying results between studies, as some found adult males had higher survival than adult females (Kuntze et al. 2024; Fogarty et al. 2022; Lofroth et al. 2023), whereas others found adult females had higher survival than adult males (Krohn et al. 1994; Gabriel et al. 2015; Sweitzer et al. 2015; Lewis et al. 2022) or little difference at all (Porteus et al. 2018). The varying geographic location and environmental conditions resulted in variation in survival among populations that researchers observed (Green, Matthews, et al. 2018). Such differences may also be due to the differences in management between the eastern populations where legal harvest is permitted and western populations where it is not (Powell 1993; Matthews et al. 2013). While not significantly different, differences between male and female survival rates were attributed to environmental characteristics or anthropogenic influences. For example, lower male survival was suggested to be linked to more movement when searching for mates, potentially exposing them to higher mortality risks. Males typically have larger home ranges and thus may also experience more risks and have reduced survival (Gabriel et al. 2015). Conversely, female fisher survival was related to the energy costs in reproduction (Sweitzer et al. 2015). Such differences in survival may also be related to sex‐specific susceptibility to harvest, as males with more movement and larger home ranges than females may be more vulnerable. Lewis et al. (2022) reported the largest differences in annual survival rates of translocated male (0.0–0.33) and female fishers (0.60–0.71), but these rates are likely greatly influenced by differences in sample sizes (*n*_male_ = 9, *n*_female_ = 51).

Although we noted differences in the variation between western and eastern survival rates, we did not find evidence that adult sex‐specific fisher survival varied between the western and eastern populations. This may indicate that while the specific mortality pressures faced by geographically disparate populations vary, the overall pressure experienced by populations is similar across their distribution. Harvest mortality may be compensatory in eastern populations for the lack of predation risk these populations face. We did observe that adult male fishers from the western populations had the greatest degree of variation in survival rates. This variation may be due to differences in the number of studies from each area (*n*_western_ = 7, *n*_eastern_ = 2), or variations in sample sizes between studies; as some studies had a single adult male fisher to monitor in a given year (Lewis et al. 2022) while others used data on > 20 adult male fishers to estimate survival (Kordosky et al. 2021; Lofroth et al. 2023). Strikingly, although there were nine studies of adult female fishers from western populations, they had less variation in survival rates.

Despite differences in study designs and methodological approaches, young fishers generally had lower survival rates than adult fishers in most of the studies across their range (Krohn et al. 1994; Sweitzer, Popescu, et al. 2016; Fogarty et al. 2022; Kuntze et al. 2024). This is because young fishers are more vulnerable to harvest and other mortality sources (Greenhorn et al. 2021), while also being dependent on their mother during the early stages of development (Powell et al. 2003). Tracking with radio collars was the most common field method for assessing fisher survival (e.g., Belant 2007; Kordosky et al. 2021; Kuntze et al. 2024). But fitting collars to juvenile mammals is often challenging as the rapid changes in body size prevent collars from being secure or risk harming the individual fitted (Kenward 2001; Casper 2009), likely reducing sample sizes for young fishers in some studies (e.g., Lofroth et al. 2023; Krohn et al. 1994). Indeed, Lofroth et al. (2023) compiled information from five radiotelemetry studies between 1990–2012 and only had 30 subadult fishers (i.e., < 2 years) compared to 70 adult fishers (i.e., > 2 years). One way researchers attempted to overcome this challenge was to trap juveniles when they were ~6 months old and had largely reached their adult body sizes (Frost and Krohn 2005). Survival was then tracked until 1 year old and adjusted based on an approximated survival for the first 6 months determined from the interactions of kit survival during the denning season and survival of adult females with trailing kits (Sweitzer, Popescu, et al. 2016).

Studies further deviated by using different classifications for age groups. While juveniles were largely defined as individuals < 12 months old (e.g., Krohn et al. 1994), not all studies applied this convention. Lewis et al. (2022) considered females < 1 year to be juveniles, whereas males < 2 years old were considered juveniles. Conversely, Lofroth et al. (2023) grouped all individuals < 2 years as subadult. Even with these varied approaches, only Lewis et al. (2022) found young fisher survival to exceed that of adult fishers. However, this study monitored far fewer adults than young fishers (e.g., 9 total adult males, 52 young males), and the differences in sample sizes likely influenced survival estimates. Our recommendation for future studies is to differentiate between juvenile and subadult fishers and attempt to obtain robust sample sizes for each age class. Other mammals exhibit different survival rates for juveniles and yearlings (Festa‐Bianchet and King 1991; Richard et al. 2014; Lehman et al. 2024), and should fishers follow a similar pattern, inferences regarding population dynamics may become biased without accounting for differences between age classes.

The few studies investigating the seasonal differences in fisher survival highlighted how survival was influenced by seasonal pressures. Both young and adult fishers had lower survival in harvest seasons than nonharvest seasons in Maine (Krohn et al. 1994), and the only fisher death reported by Belant (2007) occurred during the harvest season. These studies emphasize how anthropogenic pressure changes throughout the year. Fishers also exhibited differences in survival rates between winter and summer even in areas without legal harvest, but this varied by sex. Adult females had higher survival in winter while adult males had higher survival in summer, and researchers attributed differences to seasonal differences in prey availability and thermoregulatory or energetic needs during the fisher's life history (e.g., parturition) (Kuntze et al. 2024). However, other pressures including females defending vulnerable kits from predators or movement between denning sites may also influence female fisher survival during summer. Obstacles like radio collar failure (e.g., Krohn et al. 1994; Sweitzer et al. 2015) and small sample sizes (e.g., Belant 2007) likely contributed to the overall low number of studies assessing seasonal survival rates or examinations of finer temporal scales. Understanding these differences is a key need for future research to understand fisher population dynamics.

### 4.3 Limits of Current Knowledge

Through our literature review, we identified several important areas for future research regarding fisher mortality and survival. (1) Research into seasonal variations of cause‐specific mortality and survival would further elucidate how dynamic pressures affect fisher populations. This is because prey availability (Powell et al. 1997; Allen, Avrin, et al. 2021; Allen, Elbroch, et al. 2021), risk of predation (Wengert et al. 2014), harvest pressure (Krohn et al. 1994), and intraspecific competition (Facka and Powell 2021) vary throughout the year. (2) Further research into juvenile survival, particularly where juveniles may be impacted by harvest. While we found that many studies relied on radio collars to assess survival, the rapid growth of juveniles makes this approach difficult. Ear tagging has been a traditional alternative to radio collars or GPS, but recent advances allow researchers to use an individual's genetic data as markers for survival studies (e.g., capture–mark–recapture; Pine et al. 2013). Additionally, surgically implanted transmitters have been used to investigate fisher survival (Lewis et al. 2022; Lofroth et al. 2023) and juvenile survival in other mammals (Echols et al. 2004), potentially offering another approach to monitoring juvenile fishers. These approaches can enable researchers to assess juvenile mortality and survival without the risk of harm posed by radio collars and may be a straightforward addition to management programs where trappers are already required to submit fisher jaws for aging. However, consideration for limitations surrounding alternative approaches should be made, as the selection of methodologies for estimating survival (e.g., known‐fate, derived parameter) can impact study results. (3) Additional research into geographically underrepresented populations is also warranted, as fishers occur in many states and provinces throughout North America, but the majority of recent research is geographically concentrated across western populations (especially California). Given the differences in pressures experienced by geographically disparate populations (e.g., legal protection, predator presence, prey availability), estimates of 1 population may not be transferable to another and thereby complicate research or management that relies on such estimates. In particular, we recommend additional research investigating cause‐specific mortalities and survival of fishers in central regions (e.g., Minnesota, Montana, Idaho, Saskatchewan) and into the understudied populations at the geographical extent of their range (e.g., West Virginia).

While not included in our literature pool, we did encounter numerous studies conducted across North America; however, these were often parts of technical reports, theses, or dissertations. This suggested that research into fisher mortality and survival has been conducted in many other locations but may not be undergoing the peer‐review process. Information that ends up as gray literature has implications for fisher management and research but may not reach a larger audience (Kushkowski et al. 2003). When research is not circulated or clearly disseminated throughout the larger scientific community, efforts to compare and assess fisher populations across political boundaries or at broader spatial scales are hindered and potentially limit management efforts.

## 5 Conclusion

Wide‐ranging species with geographically disparate populations often face differing pressures across their distribution. Fishers highlight this phenomenon as they exhibit regional differences in mortality sources. Eastern fisher populations contend with legal harvest regimes, whereas western fisher populations face increased mortality risks from predation and lethal toxicant exposure. Although survival rates were not significantly different between eastern and western populations, we observed variations in survival rates between demographic groups (although small sample sizes and differing methodologies between studies complicated these comparisons). Despite numerous recent studies investigating this species, we also identified gaps in the current literature that would benefit from additional research. Such knowledge would increase our range‐wide understanding of the species and improve the conservation and management efforts centered on fishers.

## Author Contributions

**Justin J. Remmers:** conceptualization (equal), formal analysis (lead), methodology (lead), writing – original draft (lead). **Kirk W. Stodola:** funding acquisition (equal), supervision (equal), writing – review and editing (equal). **Maximilian L. Allen:** funding acquisition (equal), supervision (equal), writing – review and editing (equal).

## Conflicts of Interest

The authors declare no conflicts of interest.

## Supporting information


Table S1

## Data Availability

All data were gathered from the sources listed in Table S1.

## References

[ece371531-bib-0001] Allen, M. L. , A. C. Avrin , M. J. Farmer , et al. 2021. “Limitations of Current Knowledge About the Ecology of Grey Foxes Hamper Conservation Efforts.” Journal of Threatened Taxa 13, no. 8: 19079–19092.

[ece371531-bib-0002] Allen, M. L. , L. M. Elbroch , and H. U. Wittmer . 2021. “Scavenging by Fishers in Relation to Season and Other Scavengers.” Ecological Research 36, no. 2: 353–359. 10.1111/1440-1703.12198.

[ece371531-bib-0003] Allen, M. L. , L. A. Kammin , and L. R. LaBarge . 2024. “First Verified Documentation of a Fisher in Illinois Since the American Civil War.” Prairie Naturalist Notes 56: 15–19.

[ece371531-bib-0004] Aubry, K. B. , and J. C. Lewis . 2003. “Extirpation and Reintroduction of Fishers ( *Martes pennanti* ) in Oregon: Implications for Their Conservation in the Pacific States.” Biological Conservation 114: 79–90.

[ece371531-bib-0005] Belant, J. L. 2007. “Human‐Caused Mortality and Population Trends of American Marten and Fisher in a U.S. National Park.” Natural Areas Journal 27, no. 2: 155–160.

[ece371531-bib-0006] Bellier, E. , D. C. Ferreira , D. M. Kalb , L. S. Ganoe , A. E. Mayer , and B. D. Gerber . 2024. “A Statistical Population Reconstruction Model for Wildlife Populations: A Case Study With White‐Tailed Deer and Fisher.” Ecosphere 15: e4878.

[ece371531-bib-0007] Bender, L. C. , G. A. Schirato , R. D. Spencer , K. R. McAllister , and B. L. Murphie . 2004. “Survival, Cause‐Specific Mortality, and Harvesting of Male Black‐Tailed Deer in Washington.” Journal of Wildlife Management 68, no. 4: 743–1205.

[ece371531-bib-0008] Buckley, J. Y. , D. B. Needle , K. Royar , W. Cottrell , P. Tate , and C. Whittier . 2023. “High Prevalence of Anticoagulant Rodenticide Exposure in New England Fishers ( *Pekania pennanti* ).” Environmental Monitoring and Assessment 195: 1348. 37857759 10.1007/s10661-023-11919-x

[ece371531-bib-0009] Bulmer, M. G. 1974. “A Statistical Analysis of the 10‐Year Cycle in Canada.” Journal of Animal Ecology 43: 701–718.

[ece371531-bib-0010] Buskirk, S. W. , J. Bowman , and J. H. Gilbert . 2012. “Population Biology and Matrix Demographic Modeling of American Martens and Fishers.” In The Biology and Conservation of Martens, Sables, and Fisher: A New Synthesis, edited by K. B. Aubry , W. J. Zielinski , M. G. Raphael , G. Proulx , and S. W. Buskirk . Cornell University Press.

[ece371531-bib-0011] Casper, R. M. 2009. “Guidelines for Instrumentation of Wild Birds and Mammals.” Animal Behaviour 78: 1477–1483. 10.1016/j.anbehav.2009.09.023.

[ece371531-bib-0012] Clark, W. R. , and E. K. Fritzell . 1992. “A Review of Population Dynamics of Furbearers.” In Wildlife 2001: Populations, edited by D. R. McCollough and R. H. Barrett . Springer.

[ece371531-bib-0013] Collins, C. , and R. Kays . 2011. “Causes of Mortality in North American Populations of Large and Medium‐Sized Mammals.” Animal Conservation 14, no. 5: 474–483.

[ece371531-bib-0014] Coulter, M. W. 1960. “The Status and Distribution of Fisher in Maine.” Journal of Mammalogy 41: 1–9.

[ece371531-bib-0015] Echols, K. N. , M. R. Vaughan , and H. D. Moll . 2004. “Evaluation of Subcutaneous Implants for Monitoring American Black Bear Cub Survival.” Ursus 15, no. 2: 172–181. 10.2192/1537-6176(2004)015<0172:EOSIFM>2.0.CO;2.

[ece371531-bib-0016] Facka, A. N. , and R. A. Powell . 2021. “Intraspecific Competition, Habitat Quality, Niche Partitioning, and Causes of Intrasexual Territoriality for a Reintroduced Carnivoran.” Frontiers in Ecology and Evolution 9: 734155. 10.3389/fevo.2021.734155.

[ece371531-bib-0017] Festa‐Bianchet, M. , and W. J. King . 1991. “Effects of Litter Size and Population Dynamics on Juvenile and Maternal Survival in Columbian Ground Squirrels.” Journal of Animal Ecology 60, no. 3: 1077–1090.

[ece371531-bib-0018] Fogarty, R. D. , R. D. Weir , E. C. Lofroth , and K. W. Larsen . 2022. “Trapping Mortality Accelerates the Decline of the Fisher, an Endangered Mesocarnivore, in British Columbia, Canada.” Endangered Species Research 49: 1–12.

[ece371531-bib-0019] Frost, H. C. , and W. B. Krohn . 2005. “Postnatal Growth and Development in Fishers.” In Martens and Fishers (Martes) in Human‐Altered Environments: An International Perspective, edited by D. J. Harrison , A. K. Fuller , and G. Proulx , 253–263. Springer.

[ece371531-bib-0020] Gabriel, M. W. , L. W. Woods , R. Poppenga , et al. 2012. “Anticoagulant Rodenticides on Our Public and Community Lands: Spatial Distribution of Exposure and Poisoning of a Rare Forest Carnivore.” PLoS One 7: e40163. 22808110 10.1371/journal.pone.0040163 PMC3396649

[ece371531-bib-0021] Gabriel, M. W. , L. W. Woods , G. M. Wengert , et al. 2015. “Patterns of Natural and Human‐Caused Mortality Factors of a Rare Forest Carnivore, the Fisher ( *Pekania pennanti* ) in California.” PLoS One 10: e0140640. 26536481 10.1371/journal.pone.0140640 PMC4633177

[ece371531-bib-0022] Garant, Y. , and M. Crête . 1997. “Fisher, *Martes pennanti* , Home Range Characteristics in a High Density Untrapped Population in Southern Quebec.” Canadian Field‐Naturalist 111: 359–364.

[ece371531-bib-0023] Green, D. S. , A. N. Facka , K. P. Smith , S. M. Matthews , and R. A. Powell . 2022. “Evaluating the Efficacy of Reintroducing Fishers (*Pekania pennanti*) to a Landscape Managed for Timber Production.” Forest Ecology and Management 511: e120089.

[ece371531-bib-0024] Green, D. S. , M. E. Martin , R. A. Powell , et al. 2022. “Mixed‐Severity Wildfire and Salvage Logging Affect the Populations of a Forest‐Dependent Carnivoran and a Competitor.” Ecosphere 13: e03877.

[ece371531-bib-0025] Green, D. S. , S. M. Matthews , R. C. Swiers , et al. 2018. “Dynamic Occupancy Modelling Reveals a Hierarchy of Competition Among Fishers, Grey Foxes and Ringtails.” Journal of Animal Ecology 87, no. 3: 813–824. 10.1111/1365-2656.12791. 29282715

[ece371531-bib-0026] Green, R. E. , K. L. Purcell , C. M. Thompson , D. A. Kelt , and H. U. Wittmer . 2018. “Reproductive Parameters of the Fisher ( *Pekania pennanti* ) in the Southern Sierra Nevada, California.” Journal of Mammalogy 99: 537–553.

[ece371531-bib-0027] Greenhorn, J. E. , J. Bowmann , S. T. Denomme‐Brown , and D. M. Ethier . 2021. “Bottom‐Up Trophic Effects on Fisher *Pekania pennanti* Harvest Age Structure: Associations With Mast, Voles and Owls.” Wildlife Biology 2021, no. 4: wlb.00873.

[ece371531-bib-0028] Grilo, C. , J. A. Bissonette , and M. Santos‐Reis . 2009. “Spatial‐Temporal Patterns in Mediterranean Carnivore Road Casualties: Consequences for Mitigation.” Biological Conservation 142, no. 2: 301–313.

[ece371531-bib-0073] Helgen, K. , and F. Reid . 2018. “*Martes pennanti* (Amended Version of 2016 Assessment).” The IUCN Red List of Threatened Species 2018: e.T41651A125236220. 10.2305/IUCN.UK.2016-2.RLTS.T41651A125236220.en.

[ece371531-bib-0029] Hill, J. E. , T. L. DeVault , and J. L. Belant . 2019. “Cause‐Specific Mortality of the World's Terrestrial Vertebrates.” Global Ecology and Biogeography 28, no. 5: 680–689.

[ece371531-bib-0030] Hornocker, M. , and S. Negri . 2009. Cougar: Ecology and Conservation. University of Chicago Press. 306.

[ece371531-bib-0031] Johnson, S. A. , H. D. Walker , and C. A. Hudson . 2010. “Dispersal Characteristics of Juvenile Bobcats in South‐Central Indiana.” Journal of Wildlife Management 74, no. 3: 379–385.

[ece371531-bib-0032] Jordan, M. J. , R. H. Barrett , and K. L. Purcell . 2011. “Camera Trapping Estimates of Density and Survival of Fishers *Martes pennanti* .” Wildlife Biology 17: 266–276.

[ece371531-bib-0033] Kelly, G. M. 1977. “Fisher (*Martes pennanti*) Biology in the White Mountain National Forest and Adjacent Areas.” PhD diss., University of Massachusetts, Amherst, MA, USA.

[ece371531-bib-0034] Kenward, R. E. 2001. “Tag attachment.” In A Manual for Wildlife Radio Tagging, edited by R. E. Kenward , 123–146. Academic Press.

[ece371531-bib-0035] Koen, E. L. , J. Bowmann , and C. S. Findlay . 2007. “Fisher Survival in Eastern Ontario.” Journal of Wildlife Management 71, no. 4: 1214–1219.

[ece371531-bib-0036] Kordosky, J. R. , E. M. Gese , C. M. Thompson , et al. 2021. “Landscape of Stress: Tree Mortality Influences Physiological Stress and Survival in a Native Mesocarnivore.” PLoS One 16: e0253604. 34197517 10.1371/journal.pone.0253604 PMC8248622

[ece371531-bib-0037] Krebs, J. , E. Lofroth , J. Copeland , et al. 2004. “Synthesis of Survival Rates and Causes of Mortality in North American Wolverines.” Journal of Wildlife Management 68, no. 3: 439–741.

[ece371531-bib-0038] Krohn, W. B. , S. M. Arthur , and T. F. Paragi . 1994. “Mortality and Vulnerability of a Heavily Trapped Fisher Population.” In The Biology and Conservation of Martens, Sables, and Fishers, edited by S. W. Buskirk , A. S. Harestad , M. G. Raphael , and R. A. Powell , 137–145. Corneel University Press, 484 pp.

[ece371531-bib-0039] Kuntze, C. C. , M. Z. Peery , R. E. Green , K. L. Purcell , and J. N. Pauli . 2024. “Sex and Age Mediate the Effects of Rapid Environmental Change for a Forest Carnivore, the Fisher ( *Pekania pennanti* ).” Journal of Mammalogy 105: 13–25.

[ece371531-bib-0040] Kushkowski, J. D. , K. A. Parsons , and W. H. Wiese . 2003. “Master's and Doctoral Thesis Citations: Analysis and Trends of a Longitudinal Study.” Libraries and the Academy 3, no. 3: 459–479.

[ece371531-bib-0041] LaPoint, S. D. , J. L. Belant , and R. W. Kays . 2014. “Mesopredator Release Facilitates Range Expansion in Fisher.” Animal Conservation 18, no. 1: 50–61. 10.1111/acv.12138.

[ece371531-bib-0042] Lehman, C. P. , E. E. Morrison , B. Y. Neiles , and C. T. Rota . 2024. “Factors Influencing Population Growth in a Bobcat Population.” Journal of Wildlife Management 88, no. 4: e22561.

[ece371531-bib-0043] Lewis, J. C. 2006. Implementation Plan for Reintroducing Fishers to Olympic National Park. Washington Department of Fish and Wildlife, Wildlife Program, Olympia.

[ece371531-bib-0044] Lewis, J. C. , K. J. Jenkins , P. J. Happe , D. J. Manson , and P. C. Griffin . 2022. “Post‐Release Survival of Translocated Fishers: Implications for Translocated Success.” Journal of Wildlife Management 86, no. 3: e22192.

[ece371531-bib-0045] Lewis, J. C. , R. A. Powell , and W. J. Zielinski . 2012. “Carnivore Translocation and Conservation: Insights From Population Models and Field Data for Fishers (*Martes pennanti*).” PLoS One 7: e32726. 22479336 10.1371/journal.pone.0032726 PMC3314015

[ece371531-bib-0046] Lofroth, E. C. , C. M. Raley , J. M. Higley , et al. 2010. Conservation of Fishers (Martes pennanti) in South‐Central British Columbia, Western Washington, Western Oregon, and California‐Volume 1: Conservation Assessment. USDI Bureau of Land Management.

[ece371531-bib-0047] Lofroth, E. C. , R. D. Weir , L. R. Davis , and I. J. Hansen . 2023. “A Tale of Two Populations: Vital Rates of Fishers in British Columbia, Canada.” Journal of Wildlife Management 87: e22315.

[ece371531-bib-0049] Matthews, S. M. , J. M. Higley , K. M. Rennie , et al. 2013. “Reproduction, Recruitment, and Dispersal of Fishers ( *Martes pennanti* ) in a Managed Douglas‐Fir Forest in California.” Journal of Mammalogy 94: 100–108.

[ece371531-bib-0074] Matthews, S. M. , D. S. Green , J. M. Higley , K. M. Rennie , C. M. Kelsey , and R. E. Green . 2019. “Reproductive Den Selection and its Consequences for Fisher Neonates, a Cavity‐Obligate Mustelid.” Journal of Mammalogy 100: 1305–1316.

[ece371531-bib-0050] Munns, W. R., Jr. 2006. “Assessing Risks to Wildlife Populations Form Multiple Stressors: Overview of the Problem and Research Needs.” Ecology and Society 11, no. 1: 23.

[ece371531-bib-0051] Paragi, T. F. , W. B. Krohn , and S. M. Arthur . 1994. “Using Estimates of Fisher Recruitment and Survival to Evaluate Population Trend.” Northeast Wildlife 51: 1–11.

[ece371531-bib-0052] Pierce, B. M. , and V. C. Bleich . 2003. “Mountain Lion.” In Wild Mammals of North America: Biology, Conservation, and Management, edited by G. A. Feldhamer , B. C. Thompson , and J. A. Chapman , 2nd ed., 744–757. John Hopkins University Press.

[ece371531-bib-0053] Pine, W. E. , J. E. Hightower , L. G. Coggins , M. V. Lauretta , and K. H. Pollock . 2013. “Design and Analysis of Tagging Studies.” In Fisheries Techniques, edited by A. V. Zale , D. L. Parrish , and T. M. Sutton , 3rd ed. American Fisheries Society.

[ece371531-bib-0054] Porteus, T. A. , J. C. Reynolds , and M. K. McAllister . 2018. “Establishing Bayesian Priors for Natural Mortality Rate in Carnivore Populations.” Journal of Wildlife Management 82: 1645–1657.

[ece371531-bib-0055] Powell, R. A. 1993. The Fisher: Life History, Ecology, and Behavior. University of Minnesota Press.

[ece371531-bib-0056] Powell, R. A. , S. W. Buskirk , and W. J. Zielinski . 2003. “Fisher and Marten.” In Wild Mammals of North America: Biology, Management, and Conservation, edited by G. A. Feldhamer , B. C. Thompson , and J. A. Chapman , 635–649. John Hopkins University Press.

[ece371531-bib-0057] Powell, R. A. , and W. J. Zielinski . 1994. “Fisher.” In The Scientific Basis for Conserving Forest Carnivores, American Marten, Fisher, Lynx and Wolverine in the Western United States, edited by L. F. Ruggerio , K. B. Aubry , S. W. Buskirk , L. J. Lyon , and W. J. Zielinski , 38–73. Rocky Mountain Forest and Range Experiment Station General Technical Report RM‐254.

[ece371531-bib-0058] Powell, S. M. , E. C. York , and T. K. Fuller . 1997. “Seasonal Food Habits of Fishers in Central New England.” In Martes: Taxonomy, Ecology, Techniques, and Management, edited by G. Proulx , H. N. Bryant , and P. M. Woodard , 279–305. Provincial Museum of Alberta.

[ece371531-bib-0059] R Core Team . 2024. R: A Language and Environment for Statistical Computing. R Foundation for Statistical Computing. https://www.R‐project.org.

[ece371531-bib-0060] Richard, E. , S. E. Simpson , S. A. Medill , and P. D. McLoughlin . 2014. “Interacting Effects of Age, Density, and Weather on Survival and Current Reproduction for a Large Mammal.” Ecology and Evolution 4, no. 19: 3851–3860. 25614799 10.1002/ece3.1250 PMC4301048

[ece371531-bib-0061] Rodewald, A. , and S. Gehrt . 2014. “Wildlife Population Dynamics in Urban Landscapes.” In Urban Wildlife Conservation, edited by R. McCleery , C. Moorman , and M. Peterson . Springer. 10.1007/978-1-4899-7500-3_8.

[ece371531-bib-0062] Silveira, G. , J. L. Frair , J. Cohen , et al. 2024. “Anticoagulant Rodenticides May Affect Fisher Population Trends in the Northeastern United States.” Journal of Wildlife Management 2025: e22727. 10.1002/jwmg.22727.

[ece371531-bib-0063] Silveira, G. , J. L. Frair , L. Murphy , et al. 2024. “Drivers of Anticoagulant Rodenticide Exposure in Fishers ( *Pekania pennanti* ) Across the Northeastern United States.” Frontiers in Ecology and Evolution 12: 1304659. 10.3389/fevo.2024.1304659.

[ece371531-bib-0064] Spencer, W. , H. Rustigian‐Romsos , J. Strittholt , R. Scheller , W. Zielinski , and R. Truex . 2011. “Using Occupancy and Population Models to Assess Habitat Conservation Opportunities for an Isolated Carnivore Population.” Biological Conservation 144: 788–803.

[ece371531-bib-0065] Sweitzer, R. A. , V. D. Popescu , R. H. Barrett , K. L. Purcell , and C. M. Thompson . 2015. “Reproduction, Abundance, and Population Growth for a Fisher ( *Pekania pennanti* ) Population in the Sierra National Forest, California.” Journal of Mammalogy 96: 772–790.

[ece371531-bib-0066] Sweitzer, R. A. , V. D. Popescu , C. M. Thompson , et al. 2016. “Mortality Risks and Limits to Population Growth of Fishers: Mortality Risks and Fisher Population Growth.” Journal of Wildlife Management 80: 438–451.

[ece371531-bib-0067] Sweitzer, R. A. , C. M. Thompson , R. E. Green , R. H. Barrett , and K. L. Purcell . 2016. “Survival of Fishers in the Southern Sierra Nevada Region of California.” Journal of Mammalogy 97: 274–286.

[ece371531-bib-0068] Thomas, P. J. , K. M. Eccles , and L. J. Mundy . 2017. “Spatial Modelling of Non‐Target Exposure to Anticoagulant Rodenticides Can Inform Mitigation in Two Boreal Predators Inhabiting Areas With Intensive Oil and Gas Development.” Biological Conservation 212: 111–119.

[ece371531-bib-0069] Thompson, C. , R. Sweitzer , M. Gabriel , K. Purcell , R. Barrett , and R. Poppenga . 2014. “Impacts of Rodenticide and Insecticide Toxicants From Marijuana Cultivation Sites on Fisher Survival Rates in the Sierra National Forest, California.” Conservation Letters 7: 91–102.

[ece371531-bib-0070] United States Fish and Wildlife Service [USFWS] . 2020. “Endangered and Threatened Wildlife and Plants; Endangered Species Status for Southern Sierra Nevada Distinct Population Segment of Fisher.” Federal Register 85: 29532–29589.

[ece371531-bib-0071] Wengert, G. M. , M. W. Gabriel , S. M. Matthews , et al. 2014. “Using DNA to Describe and Quantify Interspecific Killing of Fishers in California.” Journal of Wildlife Management 78: 603–611.

[ece371531-bib-0072] Wright, P. L. , and M. W. Coulter . 1967. “Reproduction and Growth in Maine Fishers.” Journal of Wildlife Management 31, no. 1: 70–87.

