# Supplementary material for: Fisher (Pekania pennanti) Populations Exhibit Regional Differences in Cause‐Specific Mortality but Not Survival Rates
Source: Ecol Evol. 2025 Jun 5;15(6):e71531. doi: 10.1002/ece3.71531 (PMC12141758; doi:10.1002/ece3.71531)
Supplement: Supplementary file 1 — Table S1 [file ECE3-15-e71531-s001.docx]

**Supplementary Material A.**

Table A1. A literature review of peer-reviewed journal articles and book chapters containing information on fisher survival or cause-specific mortality. The review includes information on the location, sample size, and if legal harvest occurred within the study area during the study period when available. Studies investigating eastern fisher populations are shaded in grey at the bottom of the table.

| **Source** | **Location** | **Sample size** | **Legal harvest** | **Categories** |
| --- | --- | --- | --- | --- |
| Jordan et al. 2011 | California | 54 | No | Survival |
| Spencer et al. 2011 | California | NA | No | Survival |
| Gabriel et al. 2012 | California | 58 | No | Mortality |
| Matthews et al. 2013 | California | NA | No | Mortality |
| Thompson et al. 2014 | California | 46 | No | Mortality, Survival |
| Wengert et al. 2014 | California | 101 | No | Mortality |
| Gabriel et al. 2015 | California | 101 | No | Mortality |
| Sweitzer et al. 2015 | California | 167 | No | Survival |
| Sweitzer et al. 2016a | California | 232 | No | Mortality, Survival |
| Sweitzer et al. 2016b | California | 232 | No | Mortality, Survival |
| Green et al. 2018 | California, Oregon | 139 | No | Survival |
| Porteus et al. 2018 | California | NA | No | Survival |
| Matthews et al. 2019 | California | 85 | No | Survival |
| Kordorsky et al. 2021 | California | 64 | No | Survival |
| Green et al. 2022a | California | 40 | No | Survival |
| Green et al. 2022b | California | 123 | No | Survival |
| Lewis et al. 2022 | Washington | 90 | No | Mortality, Survival |
| Fogarty et al. 2022 | British Columbia | 60 | Yes | Survival |
| Lofroth et al. 2023 | British Columbia | 100 | Yes | Mortality, Survival |
| Kuntze et al. 2024 | California | 170 | No | Survival |
| Krohn et al. 1994 | Maine | NA | Yes | Mortality, Survival |
| Paragi et al. 1994 | Maine | 31 | Yes | Survival |
| Garant and Crete 1997 | Quebec | 22 | No | Survival |
| Belant 2007 | Michigan | 14 | Yes | Mortality, Survival |
| Koen et al. 2007 | Ontario | 59 | Yes | Mortality, Survival |
| Bellier et al. 2024 | Rhode Island | 503 | Yes | Survival |

**References**

Belant, J. L. 2007. Human-Caused Mortality and Population Trends of American Marten and Fisher in a U.S. National Park. Natural Areas Journal 27(2):155-160.

Bellier, E., D. C. Ferreira, D. M. Kalb, L. S. Ganoe, A. E. Mayer, and B. D. Gerber. 2024. A statistical population reconstruction model for wildlife populations: A case study with white-tailed deer and fisher. Ecosphere 15:e4878.

Fogarty, R. D., R. D. Weir, E. C. Lofroth, and K. W. Larsen. 2022. Trapping mortality accelerates the decline of the fisher, an endangered mesocarnivore, in British Columbia, Canada. Endagered Species Research 49:1-12.

Gabriel, M. W., L. W. Woods, R. Poppenga, R. A. Sweitzer, C. Thompson, S. M. Matthews, J. M. Higley, S. M. Keller, K. Purcell, R. H. Barrett, G. M. Wengert, B. N. Sacks, and D. L. Clifford. 2012. Anticoagulant Rodenticides on our Public and Community Lands: Spatial Distribution of Exposure and Poisoning of a Rare Forest Carnivore. W. M. Getz, editor. PLoS ONE 7:e40163.

Gabriel, M. W., L. W. Woods, G. M. Wengert, N. Stephenson, J. M. Higley, C. Thompson, S. M. Matthews, R. A. Sweitzer, K. Purcell, R. H. Barrett, S. M. Keller, P. Gaffney, M. Jones, R. Poppenga, J. E. Foley, R. N. Brown, D. L. Clifford, and B. N. Sacks. 2015. Patterns of Natural and Human-Caused Mortality Factors of a Rare Forest Carnivore, the Fisher (Pekania pennanti) in California. PLOS ONE 10:e0140640.

Garant, Y., and M. Crête. 1997. Fisher, Martes pennanti, home range characteristics in a high density untrapped population in southern Quebec. The Canadian field-naturalist 111:359–364.

Green, D. S., A. N. Facka, K. P. Smith, S. M. Matthews, and R. A. Powell. 2022a. Evaluating the efficacy of reintroducing fishers (Pekania pennanti) to a landscape managed for timber production. Forest Ecology and Management 511:e120089.

Green, D. S., M. E. Martin, R. A. Powell, E. L. McGregor, M. W. Gabriel, K. L. Pilgrim, M. K. Schwartz, and S. M. Matthews. 2022b. Mixed-severity wildfire and salvage logging affect the populations of a forest-dependent carnivoran and a competitor. Ecosphere 13:e03877.

Green, D.S., S. M. Matthews, R. C. Swiers, R. L. Callas, J. S. Yaeger, S. L. Farber, M. K. Schwartz, and R. A. Powell. 2018. Dynamic occupancy modelling reveals a hierarchy of competition among fishers, grey foxes and ringtails. J. Anim. Ecol. 87 (3), 813–824. https://doi.org/10.1111/1365-2656.12791.

Jordan, M. J., R. H. Barrett, and K. L. Purcell. 2011. Camera trapping estimates of density and survival of fishers Martes pennanti. Wildlife Biology 17:266–276.

Koen, E. L., J. Bowmann, and C. S. Findlay. 2007. Fisher Survival in Eastern Ontario. The Journal of Wildlife Management 71(4):1214-1219.

Kordosky, J. R., E. M. Gese, C. M. Thompson, P. A. Terletzky, L. A. Neuman-Lee, J. D. Schneiderman, K. L. Purcell, and S. S. French. 2021. Landscape of stress: Tree mortality influences physiological stress and survival in a native mesocarnivore. PLOS ONE 16:e0253604.

Krohn, W. B., S. M. Arthur, and T. F. Paragi. 1994. Mortality and vulnerability of a heavily trapped fisher population. Paes 137-145 in S. W. Buskirk, A. S. Harestad, M. G. Raphael, and R. A. Powell, eds. The biology and conservation of martens, sables, and fishers. Corneel University Press, Ithaca, NY. 484 pp.

Kuntze, C. C., M. Z. Peery, R. E. Green, K. L. Purcell, and J. N. Pauli. 2024. Sex and age mediate the effects of rapid environmental change for a forest carnivore, the Fisher (Pekania pennanti). Journal of Mammalogy 105:13–25.

Lewis, J. C., K. J. Jenkins, P. J. Happe, D. J. Manson, and P. C. Griffin. 2022. Post-release survival of translocated fishers: implications for translocated success. The Journal of Wildlife Management 86(3):e22192.

Lofroth, E. C., R. D. Weir, L. R. Davis, and I. J. Hansen. 2023. A tale of two populations: vital rates of fishers in British Columbia, Canada. The Journal of Wildlife Management 87:e22315.

Matthews, S. M., J. M. Higley, K. M. Rennie, R. E. Green, C. A. Goddard, G. M. Wengert, M. W. Gabriel, and T. K. Fuller. 2013. Reproduction, recruitment, and dispersal of fishers (Martes pennanti) in a managed Douglas-fir forest in California. Journal of Mammalogy 94:100–108.

Matthews, S. M., D. S. Green, J. M. Higley, K. M. Rennie, C. M. Kelsey, and R. E. Green. 2019. Reproductive den selection and its consequences for fisher neonates, a cavity-obligate mustelid. Journal of Mammalogy 100:1305–1316.

Paragi, T. F., W. B. Krohn, and S. M. Arthur. 1994. Using estimates of fisher recruitment and survival to evaluate population trend. Northeast Wildlife 51:1-11.

Porteus, T. A., J. C. Reynolds, and M. K. McAllister. 2018. Establishing Bayesian priors for natural mortality rate in carnivore populations. The Journal of Wildlife Management 82:1645–1657.

Spencer, W., H. Rustigian-Romsos, J. Strittholt, R. Scheller, W. Zielinski, and R. Truex. 2011. Using occupancy and population models to assess habitat conservation opportunities for an isolated carnivore population. Biological Conservation 144:788–803.

Sweitzer, R. A., C. M. Thompson, R. E. Green, R. H. Barrett, and K. L. Purcell. 2016a. Survival of fishers in the southern Sierra Nevada region of California. Journal of Mammalogy 97:274–286.

Sweitzer, R. A., V. D. Popescu, R. H. Barrett, K. L. Purcell, and C. M. Thompson. 2015. Reproduction, abundance, and population growth for a fisher (*Pekania pennanti*) population in the Sierra National Forest, California. Journal of Mammalogy 96:772-790.

Sweitzer, R. A., V. D. Popescu, C. M. Thompson, K. L. Purcell, R. H. Barrett, G. M. Wengert, M. W. Gabriel, and L. W. Woods. 2016b. Mortality risks and limits to population growth of fishers: Mortality Risks and Fisher Population Growth. The Journal of Wildlife Management 80:438–451.

Thompson, C., R. Sweitzer, M. Gabriel, K. Purcell, R. Barrett, and R. Poppenga. 2014. Impacts of Rodenticide and Insecticide Toxicants from Marijuana Cultivation Sites on Fisher Survival Rates in the Sierra National Forest, California. Conservation Letters 7:91–102.

Wengert, G. M., M. W. Gabriel, S. M. Matthews, J. M. Higley, R. A. Sweitzer, C. M. Thompson, K. L. Purcell, R. H. Barrett, L. W. Woods, R. E. Green, S. M. Keller, P. M. Gaffney, M. Jones, and B. N. Sacks. 2014. Using DNA to describe and quantify interspecific killing of fishers in California. The Journal of Wildlife Management 78:603–611.
